# Neuromuscular Blocking Agents and Monitoring in China: A Cross-Sectional Survey of Current Management

**DOI:** 10.3389/fmed.2022.770105

**Published:** 2022-04-27

**Authors:** HaoTian Wu, ZengMao Lin, RuiHao Zhou, SuiSui Huang, LingJun Chen, Yang Su, LuoNa Cheng, Huan Zhang

**Affiliations:** ^1^Department of Anesthesiology, Beijing Tsinghua Changgung Hospital, School of Clinical Medicine, Tsinghua University, Beijing, China; ^2^Department of Anesthesiology, Peking University First Hospital, School of Clinical Medicine, Peking University, Beijing, China; ^3^Department of Anesthesiology, West China Hospital, Sichuan University, Chengdu, China; ^4^Department of Anesthesiology, The Third Affiliated Hospital of Guizhou Medical University, Guiyang, China; ^5^Department of Anesthesiology, The Central Hospital of Yongzhou, School of Clinical Medicine, University of South China, Hengyang, China; ^6^Department of Anesthesiology, Kaifeng People's Hospital, Kaifeng, China; ^7^Department of Anesthesiology, Xiangdu District Hospital, Xingtai, China

**Keywords:** NMBAs, neuromuscular monitoring, residual neuromuscular block, survey, reversal

## Abstract

**Background:**

Little is known about the recent use of neuromuscular blocking agents (NMBAs) and monitoring in China. This paper presents the results of a nationwide survey conducted to obtain information regarding the current management of NMBAs in China.

**Methods:**

A questionnaire was sent to Chinese anesthesiologists inviting them to participate in the study. The questionnaire was available through the *wenjuanxing* website, and the link was sent to 1,488 anesthesiologists using the *Wechat* mini app.

**Results:**

The web-based survey consisted of 28 questions, and data were collected using an online tool. Between May 19, 2021 and June 16, 2021, 637 responses were collected (response rate = 42.8%). Only 10.2% of anesthesiologists reported using neuromuscular function monitors, and 6.59% of respondents reported that they had the relevant monitors in the operating room.

**Conclusion:**

Although PORC is a potential safety issue, the frequency of using reversal agents and monitors remains extremely low in China. Surveys such as this are important to understand the use and application customs of NMBAs in China.

## Introduction

Neuromuscular blocking agents (NMBAs) are commonly used by anesthesiologists during surgery (1). Post-operative residual curarization (PORC) is an important risk factor for anesthesia-related mortality. Even minor degrees of residual block are associated with a weakness of the upper airway muscles, airway obstruction, increased risk of aspiration, and unpleasant muscle weakness. Incomplete post-operative neuromuscular recovery can also cause prolonged recovery room stay, hypoxemia and airway obstruction, awareness during emergence from anesthesia, and increased post-operative pulmonary complications (2–4).

According to the results of a multicenter investigation in China in 2015, in a clinical series of 1,571 patients undergoing elective open or laparoscopic abdominal procedures at 32 hospitals, the incidence of PORC at the time of endotracheal extubation was 57.8% (5). Similar findings have been documented in previous studies conducted in several countries, including several developed countries. Although there are strong recommendations from guidelines and consensus statements, the residual effects of muscle relaxants and their complications have not received sufficient attention (6–12).

The aim of the survey was to evaluate the use and application of neuromuscular blocking agents and monitoring in China. An online questionnaire was designed to understand the use conventions of neuromuscular blockers.

## Methods

A cross-sectional questionnaire was sent to anesthesiologists registered in the following: Luffy Anesthesia Channel, Primary Anesthesia Network, Chinese Society of Anesthesiology, and Chinese Association of Anesthesiologists. Participants were invited via *Wechat* to complete an online survey, and encouraged to forward the invitations to colleagues. The questionnaire consisted of 28 questions, 6 investigating demographics, and 22 regarding the perioperative management of NMBAs, neuromuscular function monitoring, and antagonists. The participants accessed a link to a website for online data collection (*wjx.cn*) using the *WeChat* mini app. Data collection remained open from May 19, 2021 to June 16, 2021.

## Results

The questionnaire was sent to 1,488 anesthesiologists. During the study period, 637 responses were collected anonymously (response rate = 42.5%).

Regarding the rank of the responders, the questionnaires was answered by resident anesthesiologists (34.69%), attending anesthesiologists (43.33%), associate chief anesthesiologists (17.58%), and chief anesthesiologists (4.4%). Regarding the level of hospital, 44.43% of participants came from Grade III level A teaching hospitals (Table 1).

**Table 1:** Demographics.

**Covariate**	**Level**	**No. (%)**
Gender	Male	406 (63.74)
	Female	231 (36.26)
Age group	18~25 yrs	38 (5.97)
	26~30 yrs	139 (21.82)
	31~40 yrs	292 (45.84)
	41~50 yrs	134 (21.04)
	51~60 yrs	30 (4.71)
	>60 yrs	4 (0.63)
Work province	Anhui	24 (3.77)
	Beijing (capital)	30 (4.71)
	Chongqing	37 (5.81)
	Fujian	11 (1.73)
	Gansu	5 (0.78)
	Guangdong	35 (5.49)
	Guangxi	20 (3.14)
	Guizhou	34 (5.34)
	Hainan	5 (0.78)
	Hebei	40 (6.28)
	Heilongjiang	11 (1.73)
	Henan	45 (7.06)
	Hubei	26 (4.08)
	Hunan	19 (2.98)
	Jiangsu	26 (4.08)
	Jiangxi	15 (2.35)
	Jilin	4 (0.63)
	Liaoning	7 (1.1)
	Nei Monggol	29 (4.55)
	Ningxia	7 (1.1)
	Qinghai	2 (0.31)
	Shandong	42 (6.59)
	Shanghai	13 (2.04)
	Shanxi	18 (2.83)
	Shaanxi	17 (2.67)
	Sichuan	32 (5.02)
	Tianjin	2 (0.31)
	Xinjiang	30 (4.71)
	Yunnan	11 (1.73)
	Zhejiang	40 (6.28)
The grade and level of the hospital	A Grade III Level A hospital	283 (44.43)
where you work	A Grade III Level B hospital	79 (12.4)
	A Grade II Level A hospital	208 (32.65)
	A Grade II Level B hospital	32 (5.02)
	A Grade I hospital	16 (2.51)
	Other	19 (2.98)
Education level	High school degree	1 (0.16)
	Undergraduate degree	483 (75.82)
	Master degree	135 (21.19)
	Doctorate degree	16 (2.51)
	Postdoctoral degree	1 (0.16)
	Other	1(0.16)
Medical title	Resident physician	221 (34.69)
	Attending physician	276 (43.33)
	Associate chief physician	112 (17.58)
	Chief physician	28 (4.4)

Cisatracurium (45.68%) and rocuronium (35.48%) were the most common neuromuscular blockers used during surgery (Table 2). Only 10.2% of anesthesiologists reported using neuromuscular function monitors, and 6.59% of respondents reported that they had such monitors in the operating room. A total of 71.11% of the respondents reported using only the post-operative clinical manifestation to evaluate if the patient had recovered from the muscle relaxant. Neuromuscular blockade reversal agents, such as sugammadex and neostigmine, were used in 0.47 and 28.41% of hospitals, respectively (Table 3).

**Table 2:** Anesthesia management of neuromuscular blocking agents.

**Question**	**Answer**	**No. (%)**
When do you assess the patient's airway condition?	At pre-operative visits	569 (89.32)
	In the operating room	68 (10.68)
Do you ask the patients previous history of anesthesia?	No, I won't.	11 (1.73)
	Be sure to ask.	520 (81.63)
	Just ask if I think of it.	106 (16.64)
Do you give NMBAs during all kinds of the general anesthesia?	Yes, I will.	258 (40.5)
	Not always.	379 (59.5)
What is your order of induction?	Sedation—analgesia—NMBAs	321 (50.39)
	Sedation—NMBAs—analgesia	60 (9.42)
	NMBAs—sedation—analgesia	14 (2.2)
	NMBAs—analgesia—sedation	14 (2.2)
	Analgesia—NMBAs—sedation	54 (8.48)
	Analgesia—sedation—NMBAs	159 (24.96)
	Other combinations	15 (2.35)
Do you ventilate the patient before using NMBAs?	Unassisted ventilation	154 (24.18)
	Help to breathe	459 (72.06)
	Control breathing	24 (3.77)
Do you give the same dose before endotracheal intubation and laryngeal mask ventilation?	Not the same. give 1 to 2 times ED95 when laryngeal mask is placed	407 (63.89)
	Same, give 2~3 times ED95	125 (19.62)
	I don't give NMBAs while the laryngeal mask was ventilating	105 (16.48)
Which of the following drugs are available in your operating room?	Depolarizing muscle relaxant	6 (0.94)
	Rocuronium	226 (35.48)
	Cisatracurium	291 (45.68)
	Atracurium	16 (2.51)
	Vecuronium	98 (15.38)
When you administer the dose of NMBAs to the intubation, your rate is	1–2 s very fast	106 (16.64)
	5 s uniform	467 (73.31)
	Not <30 s turtle speed	64 (10.05)
How soon will you intubate after giving NMBAs?	In a minute	59 (9.26)
	3 min	507 (79.59)
	5 min	70 (10.99)
	Wait a minute. Take your time. Start in 10 min	1 (0.16)
How long and dose to add NMBAs during the operation?	0.5 h, 1/5~1/3 of initial dose	173 (27.16)
	1 h, 1/5 to 1/3 of the initial dose	299 (46.94)
	1 h, 1/3 to 1/2 of the initial dose	95 (14.91)
	It's too much trouble to give the drug, direct intravenous pump	70 (10.99)
About laparoscopic surgery	Deep muscle relax, 3 times ED95	222 (34.85)
	Normal muscle relax, 2 times ED95	415 (65.15)
For obese patients with BMI ≥40.0 kg/m^2^, how would you calculate the intubation dose of NMBAs?	Total body weight (TBW)	118 (18.52)
	Standard body weight (SBW) [height−80] *0.7 (male)	434 (68.13)
	Lean body weight (LBW) 1.10*TBW-0.0128*BMI*TBW (male)	85 (13.34)
If you run out of NMBAs on hand during the operation, will you switch to another kind?	Use the same muscle relaxant consistently	521(81.79)
	Thousands of choices, take your pick	116 (18.21)
When the surgery is almost over, the surgeon can't close the upper abdomen, you will choose to	Really give 1/5 to 1/3 of the initial dose of muscle relaxation	274 (43.01)
	Give the patient normal saline and tell the surgeon that muscle relaxants have been added	76 (11.93)
	Give a sedative or a deeper anesthetic	287 (45.05)

**Table 3:** Availability and use of reversal drugs and neuromuscular monitoring.

**Question**	**Answer**	**No. (%)**
After the operation, you will	Wait for the effects of drug wear off, airway protection reflex recovery then extubation	453 (71.11)
	Give neostigmine and atropine then extubated	181 (28.41)
	Give Sugammadex Sodium then extubated	3 (0.47)
Which of the following clinical signs will you evaluate before extubation?	Whether the patient is conscious, cough and swallowing reflex is restored	581 (91.07)
	Sustained head lift (5 s)	370 (57.99)
	Normal vital capacity, pattern of respiration	524 (82.13)
	PetCO_2_ and PaCO_2_ ≤ 45 mmHg	312 (48.9)
The best partner of neostigmine + Atropine	Give it at the end of the operation, whether the patient's spontaneous breathing returns or not	(8.79)
	Wait patiently for the patient's spontaneous breathing to return before giving it	581 (91.21)
Which of the following is a contraindication for the use of neostigmine?	Bronchial asthma	495 (77.59)
	Arrhythmias, especially atrioventricular block	557 (87.3)
	Myocardial ischemia, severe valve stenosis	492 (77.12)
	Mechanical intestinal obstruction	494 (77.43)
	Urinary tract infection or urinary tract obstruction	337 (52.82)
	Pregnant woman	360 (56.43)
	Allergic to bromide	425 (66.61)
Which of the following is a contraindication for the use of atropine?	Spastic palsy with brain injury in children	332 (52.04)
	Arrhythmia	464 (72.73)
	Reflux esophagitis	177 (27.74)
	The movement of the esophagus and stomach is reduced	232 (36.36)
	Glaucoma	605 (94.83)
	UC (ulcerative colitis)	168 (26.33)
	Prostatic hypertrophy and urinary tract obstruction	449 (70.38)
	CHF (congestive heart-failure)	438 (68.65)
	CHD (coronary heart disease)	427 (66.93)
	Mitral stenosis	410 (64.26)
After the muscle relaxants are administered, the patient develops skin flushing, rash, and slight changes in blood pressure and heart rate. Do you treat them?	Glucocorticoids	392 (61.54)
	Antihistamine drug	114 (17.9)
	Do not give medication and wait for symptoms to subside naturally	131 (20.57)
Do you monitor patients for NMBAs?	Yes, I do.	65 (10.2)
	No, I don't.	150 (23.55)
	There is no NMT monitors device.	380 (59.65)
	I have a NMT monitor, but I don't know how to use it.	42 (6.59)

## Discussion

The aim of this study was to evaluate the use and application of neuromuscular blocking agents and monitoring in China.

Our survey showed that only 10.2% of Chinese anesthesiologists routinely monitored muscle relaxation, a rate that is very low compared to other countries. In another survey, 80.7% of Europeans and 90.6% of Americans used neuromuscular monitors (13). 71.11% of Chinese anesthesiologists in our study judged the recovery of muscle relaxation based solely on clinical manifestations. The ability of the patient to sustain their head in an elevated position for 5 s is the most commonly used test to assess the degree of residual muscle paralysis. However, such a test cannot be considered a reliable clinical test to detect significant degrees of residual neuromuscular block (14). A very recent seminal review article underlined the relevance of monitoring neuromuscular function when using NMBAs, both in anesthesia and intensive care unit, which should be an objective and quantitative NMFM, instead of a clinical, qualitative, and subjective assessment (15). Therefore, monitoring is recommended for patients receiving NMBAs, and it is the most objective and simple way to assess the recovery of muscle relaxation after surgery. There are many reasons for the low utilization rate of muscle relaxation monitoring. On the one hand, most anesthesiologists are overconfident in their patients' clinical performance. On the other hand, because of the cost of equipping muscle relaxation monitors with each operating room and the complexity of the monitoring method, monitoring is rarely performed, and indeed, many surgery rooms lack the requisite monitors. These results indicate that popularization of muscle relaxation monitors is very important; the Chinese Society of Anesthesiology, and the Chinese Association of Anesthesiologists should publish, promote, and provide education with relevant expert consensus and guidelines on these methods.

Our questionnaire reveals that 28.88% of the respondents reported using reversal agents to reverse neuromuscular blockade. Sugammadex and neostigmine were used in 0.47% and 28.41% of hospitals, respectively. This may be due to the fact that anesthesiologists in most Chinese hospitals prefer to judge muscle relaxant metabolism based on clinical symptoms and that most hospitals are not equipped with neuromuscular monitors. In a recent study, the rates of routine use of antagonists in Europe and the United States were only 18 and 34%, respectively, and quantitative monitors were available to fewer clinicians in the United States (22.7%) than in Europe (70.2%) (*P* < 0.0001) (13). In our survey, some anesthesiologists lacked an understanding of the contraindications of neostigmine and atropine, especially atropine, which reminds us to pay extra attention to these types of patients in clinical practice. Sugammadex is a modified gamma cyclodextrin that forms a complex with the non-depolarizing NMBAs rocuronium and vecuronium (16). However, because of its high price and lack of access in Chinese health insurance, only 0.47% of our questionnaire responders used it.

The results of our survey revealed several interesting findings. Most importantly, we discovered that half of the anesthesiologists chose the sedation-analgesic-NMBAs induction order, but there were significant differences in administration speed and intubation time. Most of the responses (63.89%) indicated administering 1–2 times the ED95 when the laryngeal mask was placed. For obese patients with BMI ≥40.0 kg/m^2^, 68.13% of the responses indicated using the standard body weight (SBW) to calculate the dosage of inducible muscle relaxants. Patients undergoing laparoscopic surgery should reach a degree of deep muscle relaxation in order to prevent the abdominal pressure from being too high, to ensure good operation exposure. Studies have shown that NMBAs can improve these conditions. However, in our survey, the laparoscopic deep muscle relaxation technique was not widely used (34.85%), and when the surgeon could not close the upper abdomen during laparotomy, 45.05% of anesthesiologists chose to administer patient's normal saline while mis-informing the surgeons that muscle relaxants had been added; this may be due to concerns regarding delayed extubation or increased post-operative complications. In 2013, a French survey reported that the incidence of anaphylaxis during local or general anesthesia was ~1 in 100 (among the 1,816 cases), most of which were muscle relaxants (1,068 cases) (17). In our survey, after muscle relaxants were administered, if the patients developed skin flushing, rash, or slight changes in blood pressure and heart rate, more than half of the anesthesiologists chose intravenous glucocorticoids (61.54%).

The main limitation of our study is its small sample size; therefore, caution is warranted with regard to the generalization of the results. The nature of the questionnaire-based investigation includes a risk of data inaccuracy. Despite the small sample size, the findings would be helpful in understanding the current use of NMBAs, neuromuscular monitoring, and antagonists. Another limitation of the current survey is that no further questions were asked regarding monitoring methods and equipment for muscle relaxation. Finally, another limitation is that our survey did not enquire about serious adverse effects of muscle relaxants, including severe respiratory depression, malignant hyperthermia, and allergies.

## Conclusions

Our survey shows that NMBAs and antagonists are often administered without appropriate guidance. Most anesthesiologists are overconfident regarding their clinical manifestations. Moreover, there is poor awareness of the importance of muscle relaxant antagonist administration and monitoring.

## Data Availability Statement

The original contributions presented in the study are included in the article/Supplementary Material, further inquiries can be directed to the corresponding author/s.

## Ethics Statement

The study protocol was approved by the Beijing Tsinghua Changgung Hospital Ethics Committee (21368-6-01).

## Author Contributions

HW helped with the conceptualization, questionnaire design and validation, data curation, and original draft of the manuscript. ZL helped with investigation, provision of research background materials, and preparation of the published work. RZ helped with supervision of the execution of this national survey. SH helped with designing of this study and statistical analysis process. LJC helped with supervision of the execution of this national survey and questionnaire validation. YS helped with the conceptualization and provision of research background materials. LNC helped with provision of research background materials, manage, and coordination of this survey. HZ helped with conceptualization and reviewing of the final version of manuscript. All authors contributed to the article and approved the submitted version.

## Funding

Financial support for this study was provided, in part, by departmental funding.

## Conflict of Interest

The authors declare that the research was conducted in the absence of any commercial or financial relationships that could be construed as a potential conflict of interest.

## Publisher's Note

All claims expressed in this article are solely those of the authors and do not necessarily represent those of their affiliated organizations, or those of the publisher, the editors and the reviewers. Any product that may be evaluated in this article, or claim that may be made by its manufacturer, is not guaranteed or endorsed by the publisher.

## References

[1] Utting JE. The era of relaxant anaesthesia. Br J Anaesth. (1992) 69:551–3. 10.1093/bja/69.6.551 1467094

[2] Grosse-Sundrup M Henneman JP Sandberg WS Bateman BT Uribe JV Nguyen NT. Intermediate acting non-depolarizing neuromuscular blocking agents and risk of post-operative respiratory complications: prospective propensity score matched cohort study. BMJ. (2012) 345:e6329. 10.1136/bmj.e6329 23077290 PMC3473088

[3] Bronsert MR Henderson WG Monk TG Richman JS Nguyen JD Sum-Ping JT. Intermediate-acting nondepolarizing neuromuscular blocking agents and risk of postoperative 30-day morbidity and mortality, and long-term survival. Anesth Analg. (2017) 124:1476. 10.1213/ANE.0000000000001848 28244947

[4] Kirmeier E Eriksson LI Lewald H Jonsson Fagerlund M Hoeft A Hollmann M. Post-anaesthesia pulmonary complications after use of muscle relaxants (POPULAR): a multicentre, prospective observational study. Lancet Respir Med. (2019) 7:129–40. 10.1016/S2213-2600(18)30294-7 30224322

[5] Yu B Ouyang B Ge S Luo Y Li J Ni D. Incidence of postoperative residual neuromuscular blockade after general anesthesia: a prospective, multicenter, anesthetist-blind, observational study. Curr Med Res Opin. (2016) 32:1–9. 10.1185/03007995.2015.1103213 26452561

[6] Söderström CM Eskildsen KZ Gätke MR Staehr-Rye AK, Objective neuromuscular monitoring of neuromuscular blockade in Denmark: an online-based survey of current practice. Acta Anaesthesiol Scand. (2017) 61:619–26. 10.1111/aas.12907 28573656

[7] Pongrácz A Nemes R Breazu C Asztalos L Mitre I Tassonyi E. International survey of neuromuscular monitoring in two European countries: a questionnaire study among Hungarian and Romanian anaesthesiologists. Rom J Anaesth Intensive Care. (2019) 26:45–51. 10.2478/rjaic-2019-0007 31111095 PMC6502274

[8] Fink H Fink H Geldner G Fuchs-Buder T Hofmockel R Blobner M. [Muscle relaxants in Germany 2005: a comparison of application customs in hospitals and private practices]. Anaesthesist. (2006) 55:668–78. 10.1007/s00101-006-1015-6 16609885

[9] Cammu GV Klewais LR Vandeput DM Foubert LA. Neuromuscular monitoring, reversal and postoperative residual neuromuscular block: an intradepartmental survey over the years. Anaesth Intensive Care. (2020) 48:73–5. 10.1177/0310057X19897655 31979984

[10] Nauheimer D Fink H Fuchs-Buder T Geldner G Hofmockel R Ulm K. Muscle relaxant use for tracheal intubation in pediatric anaesthesia: a survey of clinical practice in Germany. Paediatr Anaesth. (2009) 19:225–31. 10.1111/j.1460-9592.2008.02803.x 19175884

[11] Bouderka MA Nsiri A Bouhouri A Bouaggad A Alharrar R Hamoudi D. [Moroccan survey about neuromuscular relaxant blocking drugs use and reversal management]. Ann Fr Anesth Reanim. (2014) 33:21–5. 10.1016/j.annfar.2013.06.018 24440733

[12] Locks Gde F Cavalcanti IL Duarte NM da Cunha RM de Almeida MC. Use of neuromuscular blockers in Brazil. Braz J Anesthesiol. (2015) 65:319–25. 10.1016/j.bjane.2015.03.001 26323727

[13] Naguib M Kopman AF Lien CA Hunter JM Lopez A Brull SJ. A survey of current management of neuromuscular block in the United States and Europe. Anesth Analg. (2010) 111:110–9. 10.1213/ANE.0b013e3181c07428 19910616

[14] Naguib M Brull SJ Kopman AF Hunter JM Fülesdi B Arkes HR. Consensus statement on perioperative use of neuromuscular monitoring. Anesth Analg. (2018) 127:71–80. 10.1213/ANE.0000000000002670 29200077

[15] Naguib M Brull SJ Hunter JM Kopman AF Fülesdi B Johnson KB. Anesthesiologists' overconfidence in their perceived knowledge of neuromuscular monitoring and its relevance to all aspects of medical practice: an international survey. Anesth Analg. (2019) 128:1118–26. 10.1213/ANE.0000000000003714 31094776

[16] Asztalos L Szabó-Maák Z Gajdos A Nemes R Pongrácz A Lengyel S. Reversal of vecuronium-induced neuromuscular blockade with low-dose sugammadex at train-of-four count of four: a randomized controlled trial. Anesthesiology. (2017) 127:441–9. 10.1097/ALN.0000000000001744 28640017

[17] Anaphylactic reactions during anaesthesia: neuromuscular blocking agents latex and antibiotics. Prescrire Int. (2013) 22:123–4. 23819173

